# Brazilian Society of Otology task force – single sided deafness – recommendations based on strength of evidence

**DOI:** 10.1016/j.bjorl.2024.101514

**Published:** 2024-09-24

**Authors:** Robinson Koji Tsuji, Rogério Hamerschmidt, Joel Lavinsky, Felippe Felix, Vagner Antonio Rodrigues Silva

**Affiliations:** a Universidade de São Paulo (USP), Faculdade de Medicina, Departamento de Otorrinolaringologia, São Paulo, SP, Brazil; b Universidade Federal do Paraná (UFPR), Departamento de Otorrinolaringologia, Curitiba, PR, Brazil; c Universidade Federal do Rio Grande do Sul (UFRGS), Departamento de Ciências Morfológicas, Porto Alegre, RS, Brazil; d Universidade Federal do Rio de Janeiro (UFRJ), Hospital Universitário Clementino Fraga Filho (HUCFF), Rio de Janeiro, RJ, Brazil; e Universidade Estadual de Campinas (Unicamp), Faculdade de Ciências Médicas (FCM), Departamento de Otorrinolaringologia, Cirurgia de Cabeça e Pescoço, Campinas, SP, Brazil

**Keywords:** Single-sided deafness, Cochlear implants, Contralateral routing of signal devices, Bone conduction devices, Hearing loss

## Abstract

**Objective:**

To make evidence-based recommendations for the treatment of Single-Sided Deafness (SSD) in children and adults.

**Methods:**

Task force members were instructed on knowledge synthesis methods, including electronic database search, review and selection of relevant citations, and critical appraisal of selected studies. Articles written in English or Portuguese on SSD were eligible for inclusion. The American College of Physicians’ guideline grading system and the American Thyroid Association’s guideline criteria were used for critical appraisal of evidence and recommendations for therapeutic interventions.

**Results:**

The topics were divided into 3 parts: (1) Impact of SSD in children; (2) Impact of SSD in adults; and (3) SSD in patients with temporal bone tumors.

**Conclusions:**

Decision-making for patients with SSD is complex and multifactorial. The lack of consensus on the quality of outcomes and on which measurement tools to use hinders a proper comparison of different treatment options. Contralateral routing of signal hearing aids and bone conduction devices can alleviate the head shadow effect and improve sound awareness and signal-to-noise ratio in the affected ear. However, they cannot restore binaural hearing. Cochlear implants can restore binaural hearing, producing significant improvements in speech perception, spatial localization of sound, tinnitus control, and overall quality of life. However, cochlear implantation is not recommended in cases of cochlear nerve deficiency, a relatively common cause of congenital SSD.

## Highlights

• CND and cCMV should be prioritized in the investigation of congenital SSD.
• CI is not recommended in children with SSD due to CND.
• CI is the most effective treatment for restoring hearing in SSD.
• Insufficient evidence to determine auditory deprivation duration for CI in SSD.

## Introduction

Single-Sided Deafness (SSD) is a condition currently characterized by 2 definitions: 1) Severe-to-profound hearing loss (Pure Tone Average [PTA > 70 dB]) in one ear and Normal Hearing (NH) thresholds (PTA ≤ 30 dB) assessed by pure tone audiometry in the contralateral ear^1^ or 2) Absence of hearing improvement with conventional hearing aids in the poorer ear and PTA of 20 dB HL or better in the contralateral ear.^2^ The lack of a consistent definition and confusion over nomenclature have made it difficult to clearly establish the prevalence of SSD.

The impact of hearing loss on global health is becoming increasingly recognized, but SSD-related disabilities are often underestimated. Binaural hearing is compromised by profound Unilateral Hearing Loss (UHL), reducing an individual’s ability to localize sounds and process speech in noisy environments, with consequent auditory and social implications, and eventually having an impact on academic performance.^3^, ^4^ The negative educational and professional consequences also result in reduced job opportunities and a decreased perception of Quality Of Life (QOL).^5^

The ability to locate and differentiate sound sources is important for most species, playing a crucial role in guiding behavioral responses such as pursuing potential mates or prey and avoiding or escaping from approaching predators, especially if the sound source lies beyond the detection range of the other senses, either because it is located outside the visual field or is too far away to be registered by other sensory receptors. Directional hearing is primarily based on the fact that animals have 2 ears that are physically separated on either side of the head, or in the case of some insects, on other parts of the body. That is, depending on the location of the sound source, the signals reaching each ear may differ in their time of arrival or intensity, giving rise to binaural spatial cues.^6^

Binaural cues are essential for localizing sound sources and improving the perception of target sounds in the presence of other interfering sounds.^7^ By eliminating binaural cues, or at least altering the relationship between the interaural acoustic differences and directions in space, unilateral or asymmetric hearing loss may have deleterious effects on spatial hearing. Additionally, monaural deprivation in childhood can induce maladaptive changes in the brain that may persist even if hearing in the affected ear is restored, resulting in long-term deficits in spatial hearing.^8^ However, there is growing evidence that plasticity of central auditory processing may help to partially compensate for hearing loss in one ear, leading to some recovery in the ability to localize sound.^9^

In addition to providing a basis for sound localization, the ability to extract interaural information facilitates target detection in noisy environments, a phenomenon known as the squelch effect.^10^ This refers to the change in speech reception (or target detection) thresholds in the presence of interfering sounds when the target and masking sounds are spatially separated. Spatial release from masking is a process that can support auditory stream segregation, including the ability to perceive a particular speaker’s voice in competing-noise situations, where other interfering sounds are simultaneously present.^11^

Animal studies have examined the effects of UHL during development on the morphology, connectivity, and response properties of neurons at multiple levels of the auditory system.^9^, ^12^, ^13^ The results of most of these studies are consistent with UHL causing a weakening of the representation of the deprived ear and a strengthening of the representation of the intact ear. Likewise, chronic stimulation of one ear via a Cochlear Implant (CI) during early life has been shown to result in a pronounced reorganization of cortical responses in humans and animals in favor of the stimulated ear.^14^, ^15^

Options for hearing rehabilitation in patients with SSD include hearing aids with Contralateral Routing of Signal (CROS) systems, Bone Conduction Devices (BCDs), and CIs. Variable results have been demonstrated for speech perception, speech recognition in noise, and sound localization with these devices.^5^, ^16^, ^17^, ^18^, ^19^ The choice of the most suitable device will depend on each patient.

## Objective

To make evidence-based recommendations for the treatment of SSD in children and adults.

## Methods

The Brazilian Society of Otology (Sociedade Brasileira de Otologia, SBO) met some members to discuss the topic of this guideline. Each author was asked to write a text with the current literature on the topic, based on evidence and containing the elements discussed during the meeting. A rapporteur prepared the final text, which was reviewed by additional coauthors and the Brazilian Journal of Otorhinolaryngology editor.

This guideline is not intended to be a substitute for individual professional judgment. Physicians should always act and decide in a way that they believe is best for their patients, regardless of guideline recommendations. They should also operate within their scope of practice and in accordance with their training. The guidelines represent the best judgment of a team of experienced physicians addressing the scientific evidence for a given topic.

The grading system of the American College of Physicians (ACP) was used in this guideline, relating to critical appraisal and recommendations on therapeutic interventions (Table 1, Table 2).^20^ An important component of this guideline was judged to be critical appraisal of diagnostic testing studies. However, the ACP guideline grading system was not designed for this purpose.^21^, ^22^, ^23^

**Table 1:** Interpretation of the American College of Physicians’ Guideline grading system (for therapeutic interventions).

Recommendation	Clarity of risk/benefit	Implications
Strong recommendation	Benefits clearly outweigh harms and burdens, or vice versa.	Patients: Most would want course of action; a person should request discussion if an intervention is not offered.
		Clinicians: Most patients should receive the recommended course of action.
		Policymakers: The recommendation can be adopted as policy in most circumstances.
Weak recommendation	Benefits closely balanced with harms and burdens.	Patients: Many would want course of action, but some may not; the decision may depend on individual circumstances.
		Clinicians: Different choices will be appropriate for different patients; the management decision should be consistent with patients’ preferences and circumstances.
		Policymakers: Policymaking will require careful consideration and stakeholder input.
No recommendation	Balance of benefits and risks cannot be determined.	Decisions based on evidence cannot be made.

**Table 2:** Recommendations (for therapeutic interventions) based on strength of evidence.

Recommendation and evidence of quality	Description of supporting evidence^a^	Interpretation
Strong recommendation		
High-quality evidence	RCT without important limitations or overwhelming evidence from observational studies	Can apply to most patients in most circumstances without reservation
Moderate-quality evidence	RCT with important limitations or strong evidence from observational studies	Can apply to most patients in most circumstances without reservation
Low-quality evidence	Observational studies/case studies	May change when higher-quality evidence becomes available
Weak recommendation		
High-quality evidence	RCT without important limitations or overwhelming evidence from observational studies	Best action may differ based on circumstances or patients’ values
Moderate-quality evidence	RCT with important limitations or strong evidence from observational studies	Best action may differ based on circumstances or patients’ values
Low-quality evidence	Observational studies/case studies	Other alternatives may be equally reasonable
Insufficient	Evidence is conflicting, of poor quality, or lacking	Insufficient evidence to recommend for or against

RCT, multicenter Randomized Controlled Trial.

a This description of supporting evidence refers to therapy, therapeutic strategy, or prevention studies. The description of supporting evidence is different for diagnostic accuracy studies.

The American Thyroid Association (ATA) created a diagnostic test appraisal system that used the following methodological elements: consecutive recruitment of patients representative of clinical practice, use of an appropriate reference gold standard, directness of evidence (target population of interest, testing procedures representative of clinical practice, and relevant outcomes), precision of diagnostic accuracy measures (confidence intervals for estimates such as sensitivity and specificity), and consistency of results across studies using the same test that was also used in this guideline (Table 3, Table 4).^22^

**Table 3:** Interpretation of the American Thyroid Association guideline for diagnostic tests.

Recommendation	Accuracy of diagnostic information vs. risks and burden of testing	Implications
Strong recommendation	Knowledge of the diagnostic test result clearly outweighs risks and burden of testing or vice versa.	Patients: In the case of an accurate test for which benefits outweigh risks/burden, most would want the diagnostic to be offered (with appropriate counseling). A patient should request discussion of the test if it is not offered. In contrast, for a test in which risks and burden outweigh the benefits, most patients should not expect the test to be offered.
		Clinicians: In the case of an accurate test for which benefits outweigh risks/burden, most patients should be offered the diagnostic test (and provided relevant counseling). Counseling about the test should include a discussion of the risks, benefits, and uncertainties related to testing (as applicable), as well as the implications of the test result. In contrast, for a test in which risks and burden outweigh the perceived benefits, most patients should not be offered the test, or if the test is discussed, the rationale against the test should, for the particular clinical situation, be explained.
		Policymakers: In the case of an accurate test for which benefits outweigh risks/burden, availability of the diagnostic test should be adopted in health policy. In contrast, for a test in which risks and burden outweigh the perceived benefits, some restrictions on circumstances for test use may need to be considered.
Weak recommendation	Knowledge of the diagnostic test result is closely balanced with risks and burden of testing.	Patients: Most would want to be informed about the diagnostic test, but some would not want to seriously consider undergoing the test; a decision may depend on the individual circumstances (e.g., risk of disease, comorbidities, or other), the practice environment, feasibility of optimal execution of the test, and consideration of other available options.
		Clinicians: Different choices will be appropriate for different patients, and counseling about the test (if being considered) should include a discussion of the risks, benefits, and uncertainties related to testing (as applicable), as well as the implications of the test result. The decision to perform the test should include consideration of the patients’ values, preferences, feasibility, and the specific circumstances. Counseling the patient on why the test may be helpful or not, in her/his specific circumstance, may be highly valuable in the decision-making process.
		Policymakers: Policymaking decisions on the availability of the test will require discussion and stakeholder involvement.
No recommendation	Balance of knowledge of the diagnostic test result cannot be determined.	Decisions on the use of the test based on evidence from scientific studies cannot be made.

**Table 4:** Recommendations (for diagnostic interventions) based on strength of evidence.

Recommendation and evidence of quality	Methodological quality of supporting evidence	Interpretation
Strong recommendation		
High-quality evidence	Evidence from one or more well-designed nonrandomized diagnostic accuracy studies (i.e., observational ‒ cross-sectional or cohort) or systematic reviews/meta-analyses of such observational studies (with no concern about internal validity or external generalizability of the results)	Implies the test can be offered to most patients in most applicable circumstances
Moderate-quality evidence	Evidence from nonrandomized diagnostic accuracy studies (cross-sectional or cohort), with one or more possible limitations causing minor concern about internal validity or external generalizability of the results	Implies the test can be offered to most patients in most applicable circumstances without reservation
Low-quality evidence	Evidence from nonrandomized diagnostic accuracy studies with one or more important limitations causing serious concern about internal validity or external generalizability of the results	Implies the test can be offered to most patients in most applicable circumstances, but the utilization of the test may change when higher-quality evidence becomes available.
Weak recommendation		
High-quality evidence	Evidence from one or more well-designed nonrandomized diagnostic accuracy studies (i.e., observational ‒ cross-sectional or cohort) or systematic reviews/meta-analyses of such observational studies (with no concern about internal validity or external generalizability of the results)	The degree to which the diagnostic test is seriously considered may differ depending on circumstances or patients’ or societal values
Moderate-quality evidence	Evidence from nonrandomized diagnostic accuracy studies (cross-sectional or cohort), with one or more possible limitations causing minor concern about internal validity or external generalizability of the results	The degree to which the diagnostic test is seriously considered may differ depending on individual patients’/practice circumstances or patients’ or societal values
Low-quality evidence	Evidence from nonrandomized diagnostic accuracy studies with one or more important limitations causing serious concern about internal validity or external generalizability of the results	Alternative options may be equally reasonable.
Insufficient	Evidence may be of such poor quality, conflicting, lacking (i.e., studies not done), or not externally generalizable to the target clinical population such that the estimate of the true effect of the test is uncertain and does not permit a reasonable conclusion to be made.	Insufficient evidence exists to recommend for or against routinely offering the diagnostic test.

## Results

### Unilateral hearing loss in children

UHL has an incidence of 0.4–3.4 cases per 1000 live births and a prevalence in school-aged children of 3%‒6%, increasing to 15% in children aged 6 to 19-years.^24^ The incidence of SSD is estimated to be between 2 and 5 per 1000 school-aged children.^25^ UHL affects a child’s behavior, speech and language development, and educational performance.^26^, ^27^ Children with UHL experience difficulty recognizing speech in noise,^28^, ^29^, ^30^ localizing sound sources,^18^, ^31^, ^32^ and recognizing speech in quiet^28^, ^29^ compared with children with NH. Children with SSD have lower intelligence quotients than their peers with NH.^27^, ^33^ Fatigue occurs due to the child’s increased effort to listen.^34^ Difficulties may be increased for school-aged children in noise-rich environments.^35^, ^36^

The ability of the auditory system to extract spatial information depends mainly on the detection and interpretation of binaural cues, that is, differences in the time of arrival or level of the sound between the 2 ears. UHL during development weakens the brain’s representation of the deprived ear, and this may outlast the restoration of function in that ear and therefore impair performance on tasks such as sound localization and spatial release from masking that rely on binaural processing. However, UHL also triggers a reweighting of the cues used for sound localization, resulting in increased reliance on the spectral cues provided by the other ear for azimuth localization, as well as adjustments in binaural sensitivity that help to offset the imbalance in inputs between the ears. These adaptive strategies allow the developing auditory system to largely compensate for asymmetric hearing loss while maintaining accurate sound localization. The ability of the auditory system to undergo these adaptive changes has important implications for rehabilitation strategies in those with hearing impairment.^6^

Spatial hearing skills emerge over time. Development impacts speech recognition skills^29^, ^32^, ^37^ and sound source localization.^18^, ^38^ Children may not achieve adult-like masked speech recognition scores until they approach adolescence due to challenging hearing conditions.^29^, ^32^, ^37^ Localization matures earlier, with adult-like performance typically achieved by 5–6 years of age.^39^

The true impact of SSD in children is often underestimated. As many of these children are able to understand spoken language well in quiet environments with an NH ear, they are often mistakenly perceived to hear normally by caregivers. However, there is substantial evidence of the difficulty these children face when listening in challenging conditions such as background noise (e.g., in school classrooms and social gatherings). Furthermore, SSD has been shown to contribute to delays in speech and language acquisition, impaired educational progress, and an increased incidence of behavioral problems.^40^, ^41^, ^42^, ^43^ Performance declines are likely due to the head shadow effect and the absence of central binaural integration cues, including squelch and summation.

Auditory deprivation resulting from monaural sensory input in children with SSD may have a significant impact on the development of auditory pathways and brain networks associated with cognitive functions.^44^, ^45^ Children with congenital or early-onset SSD lack access to the cues that are needed to develop these skills.^3^ This results in an inability to benefit from binaural summation or squelch and in a limited ability to benefit from the head shadow effect. Binaural summation is the benefit associated with access to the same target speech information from both ears. Binaural squelch occurs when timing and level differences between the ears provide cues that support selective listening based on binaural difference cues. The head provides a barrier that may result in a reduced masker level in one ear when the noise source is in the contralateral side. This results in an improved target-to-masker ratio in the ear within the head shadow, a cue not accessible in the deaf ear of patients with SSD.^46^

For individuals with NH, masked speech recognition improves when target and masker are separated as compared to when they are colocated on the horizontal plane.^46^ This benefit, known as Spatial Release from Masking (SRM), is not observed in listeners with SSD when the masker is in the better hearing ear. Listeners with SSD may benefit from head shadow and have SRM when the masker is in the deaf ear, but this benefit depends on monaural (as opposed to binaural) cues.

Another spatial hearing skill that is impaired in listeners with SSD is sound source localization. Children with NH use timing and level differences to locate where sound is coming from on a horizontal plane. Low frequencies provide most of the interaural timing differences between the ears, allowing the listener to determine which side of the head the sound arrived at first. Higher frequencies provide interaural level cues due to the head shadow effect. With monaural listening, children with SSD are not able to use any of these interaural difference cues to localize sound.^6^

Children with SSD aged 9 to 14-years have difficulty with auditory processing.^47^ Children with SSD have different patterns of functional connectivity responsible for auditory and executive functions, which may explain behavioral and educational difficulties. Resting-state functional connectivity Magnetic Resonance Imaging (rs-fcMRI) studies have shown that, in the cortical networks supporting executive functions in children with UHL, there are areas that have both adaptive (i.e., strengthened) and maladaptive (i.e., weakened) functional cortical networks, with a lack of predefined suppression in these networks.^48^ These findings provide a possible explanation for the educational difficulties experienced by children with UHL. While studies are scarce and there is bias in enrollment and etiology, there appears to be a direct link between UHL and cognitive development. QOL also appears to be impaired in children and adolescents with SSD in domains related to school performance and social interaction compared with their peers with NH.^49^

Congenital SSD may be secondary to Cochlear Nerve Deficiency (CND), Cytomegalovirus (CMV) and mumps infection, and inner ear abnormalities. Only a small number of early-onset SSD cases are attributable to genetic causes (Waardenburg syndrome), which are most often associated with bilateral sensorineural hearing loss.^50^ Children with UHL may progress to bilateral deafness. Fitzpatrick et al.^51^ reported that, of 537 children diagnosed with SSD at birth, 42.2% experienced hearing deterioration over the years, with 19% of cases progressing to bilateral deafness.

#### Congenital CMV (cCMV)

The worldwide prevalence rate of cCMV infection ranges from 0.2% to 2.5%.^52^, ^53^ Among all infected newborns, 80%‒90% will be asymptomatic.^54^, ^55^ Approximately 10% of asymptomatic newborns will develop hearing impairment, with the majority having severe or profound levels of impairment. Approximately 42.6% of these patients will require hearing rehabilitation.^53^ Infection with cCMV may lead to a wide spectrum of dysfunctions that, in addition to sensorineural hearing loss, include blindness and neurodevelopmental delay.^56^

Time to the diagnosis of cCMV is short, approximately 2 to 3-weeks after birth. Available tests become undefined after this period. Failing the Universal Newborn Hearing Screening (UNHS) is twice as common in newborns with cCMV as in uninfected newborns.^57^ Hearing loss is 90 times more frequent in children infected with cCMV than in uninfected children. One-third of cases of bilateral sensorineural hearing loss and half of cases of UHL are associated with cCMV.

Sensorineural hearing loss occurred in 9.9% of asymptomatic children and 33% of symptomatic cases according to a systematic review conducted in 2014.^53^ Foulon et al.^58^ demonstrated that the incidence of sensorineural hearing loss in asymptomatic infected individuals is 22% and that 5% of the children had late-onset deafness at 10-years of follow-up. Fowler et al.^59^ showed that up to 40% of asymptomatic children with congenital infection had sensorineural hearing loss, with a late-onset sensorineural hearing loss rate of up to 50% in this group (mean onset at 44-months).

Asymptomatic disease can lead to silent lesions in the inner ear structures that may progress in severity over time, resulting in late-onset hearing loss.^55^ In a longitudinal study of 651 asymptomatic children, Dahle et al.^55^ found that 37.5% developed late-onset hearing loss at a median age of 44-months. Approximately 54% experienced progressive, fluctuating hearing loss in at least one frequency at a median age of 51-months. Approximately 10% of asymptomatic cases progressed to hearing loss over 48-months.

The diagnosis of cCMV is made by identifying viral particles in saliva and urine using a Polymerase Chain Reaction (PCR) technique up to the child’s third week of life,^60^ as sensitivity decreases over the days. In intrauterine life, after 20-weeks’ gestation, identification can be done via the amniotic fluid. This procedure is indicated in very specific situations. A PCR test for CMV in amniotic fluid can confirm cCMV infection (positive predictive value is close to 100%).^61^

While Dried Blood Spot (DBS)-PCR assays are used in newborn screening for other congenital diseases, their use in cCMV screening has shown low sensitivity (28%‒75%) in prospective studies compared with gold standard methods for detecting viral antigens in saliva and urine.^61^, ^62^ A meta-analysis of 15 studies evaluated the performance of DBS-PCR assays used in cCMV screening and found a sensitivity of 62.3%.^63^ In cCMV infection, which has a low prevalence (0.2%‒2% of live births), low screening sensitivity is a limiting factor for the use of these assays.

PCR testing is most often performed in patients considered a target for the disease, such as symptomatic patients with a suggestive clinical status.^60^, ^64^ Children who fail the UNHS or who present with sensorineural hearing loss of unapparent cause should be tested for cCMV regardless of age.^60^, ^64^ The same applies to neonates born to mothers with a documented seroconversion during pregnancy. According to Cannon et al.,^65^ for cases of cCMV-induced sensorineural hearing loss, there is good evidence that routine newborn screening has a positive impact on communication-related outcomes and should be considered for all children.^60^, ^64^

In children undergoing etiologic evaluation of sensorineural hearing loss over 3 weeks of age, cCMV infection can only be confirmed retrospectively, using stored newborn blood as a model source for PCR-based diagnosis. If cCMV infection is suspected, in addition to laboratory testing, brain imaging (cranial ultrasound or MRI), visual function assessment, and hearing assessment are required.^57^, ^66^

Common findings suggestive of cCMV include central nervous system malformations, ventriculomegaly, microcephaly, and cortical and periventricular calcifications. Because these changes are present in 80% of patients with sensorineural hearing loss and symptomatic cCMV,^58^ they are relevant for the otolaryngologist providing hearing rehabilitation for these patients. Those with the most limited changes have demonstrated the best rehabilitation outcomes.

#### Cochlear nerve hypoplasia

Given the impact of UHL and SSD on development, imaging should be performed to better assess the inner ear and guide clinical management decisions. Computed Tomography (CT) of the temporal bone or MRI of the brain are most commonly performed. Malformations have been diagnosed by CT and/or MRI in 25% to 67% of patients with UHL,^67^, ^68^ with rates ranging from 3% to 50% in the literature evaluating only patients with SSD.^67^

CND presents on a spectrum ranging from complete aplasia to hypoplasia. The cochlear nerve is best assessed using high-resolution T2-weighted MRI,^69^ which has increased the rates of CND diagnosis. The cochlear nerve may be deficient on MRI even when there is a normal cochlear aperture or Internal Auditory Canal (IAC).^70^, ^71^

While several studies have assessed CND in pediatric patients with UHL, the literature has reported widely variable prevalence of CND ranging from 9% to 50%.^68^, ^70^, ^72^, ^73^ Assessment of CND in pediatric patients with UHL has been limited in the literature, with a range of 0%‒28% prevalence of CND.^73^, ^74^ Of note, studies comparing the prevalence of CND in pediatric vs bilateral SSD have demonstrated significantly higher rates of CND in UHL (39%‒50%) than in bilateral hearing loss (5%).^75^, ^76^

Assessment of hearing thresholds in patients with CND has also been limited in the literature. Patients with CND have profound hearing loss or no response on unaided or aided threshold testing.^77^, ^78^, ^79^ However, cases have been reported of ears with CND on imaging that demonstrate mild-to-moderate or moderate hearing loss.^77^, ^80^ These reports suggest that hearing thresholds alone may not be a good predictor of good cochlear nerve integrity.

The diagnosis of CND has a significant impact on the clinical management of pediatric patients with SSD. The prognosis for open-set speech perception in ears with CND is poor compared with ears with normal nerves. However, many ears with CND demonstrate a benefit from CI surgery, and some even achieve speech recognition.^78^, ^79^, ^81^, ^82^ Implanted ears with cochlear nerve hypoplasia achieve more positive outcomes than those with cochlear nerve aplasia.

Outcomes in patients with aplastic nerves typically show very limited sound perception with a CI, although there have been reports of post-implant speech recognition.^83^, ^84^ In addition to traditional bilateral candidates, CI also provides benefits to pediatric patients with SSD.^85^ Because CI is contraindicated in cases of UHL or SSD with CND and outcomes may be more limited with certain types of inner ear malformations, there is a need to recognize how common CND and cochlear malformations are in this population. This will in turn allow for a more accurate determination of CI candidacy by alerting providers to the high risk of CND or malformations in this population.

#### Imaging in children with SSD

While studies have emphasized the need for MRI to identify CND in contrast to CT, MRI is generally associated with higher cost, less availability, and longer image acquisition time.^73^, ^86^, ^87^ Furthermore, in pediatric populations, MRI may require procedural sedation or general anesthesia, which carries a possible risk of adverse neurodevelopmental effects, particularly in very young children.^88^ As such, there is benefit in identifying CT findings that can reliably predict CND.

One such finding is the width of the cochlear aperture, also known as the bony cochlear nerve canal, which can be stenotic in patients with UHL.^89^ The normal width of the cochlear aperture is estimated to be approximately 1.9 mm (SD = 0.24 mm) on CT, and the normal width of the cochlear nerve at the fundus of the IAC is estimated to be approximately 1.2 mm (SD = 0.2 mm).^90^ While there is no established cutoff value for CND, recent studies have shown a strong relationship between CND and cochlear aperture stenosis (CAS, defined as cochlear aperture widths ranging from <1.4 to <1.7 mm), and this suggests that CAS may be a predictor of CND.^72^, ^91^

Dorismond et al.^92^ identified CND in 98.5% (64/65) of ears with CAS using a cutoff of < 1.4 mm. Komatsubara et al.^93^ found that 9/10 (90%) patients with cochlear aperture width <1.5 mm had CND, while Tahir et al.^91^ found CND in 53/59 (89%) patients with cochlear aperture width <1.6 mm. Clemmens et al.,^72^ based on MRI alone, determined that 27/29 (93.1%) patients with cochlear aperture width <1.7 mm had CND.

Some authors advocate for initial MRI due to the absence of radiation exposure, its ability to identify additional brain and cranial nerve abnormalities, and its status as the gold standard for detection of CND, which dictates CI candidacy.^72^, ^94^ Others recommend obtaining an initial CT scan because of the high likelihood of yielding positive findings, possibility of identifying relevant anatomic variants, rapid image acquisition, cost-effectiveness, and feasibility of being performed without sedation.^95^ Ultimately, the decision for the optimal initial imaging modality requires consideration of multiple factors.

Patient comorbidities should also be considered when deciding on the best initial imaging modality. In patients with syndromes such as CHARGE syndrome, Trisomy 21 or 18, and VACTERL/VATER syndrome (an acronym for a disorder that can affect different organs and systems: V ‒ the Vertebrae [bones of the spinal column]; A ‒ Anus; C ‒ Cardiac anomalies; T ‒ Trachea; E ‒ Esophagus; R ‒ Renal [kidney] anomalies; L ‒ Limb differences), MRI is preferred to CT.^92^

In the absence of these patient-related factors, however, CT should be strongly considered for initial imaging. A meta-analysis of imaging features in patients with UHL found that CAS was the most common CT abnormality observed in patients with isolated UHL, present in approximately 44%.^96^ While there are cases in which the cochlear nerve can be deficient in the absence of CAS warranting subsequent MRI,^91^, ^97^ an initial CT diagnosis of CAS may be predictive of CND and provide adequate information to determine CI candidacy in patients with UHL.

Shared decision-making should include a conversation about patient-specific factors as well as the risks and benefits of each imaging modality. In cases in which CT is initially obtained and CAS is observed, CT likely provides adequate information to determine CI candidacy and obviates the need for MRI to directly assess CND.^98^ This approach may ultimately lead to reduced cost, more timely diagnosis, and avoidance of sedation in these patients.

Recommendations

I Children with a diagnosis of SSD should receive special attention. In the classroom, they should sit closer to the teacher, with their normal ear facing the teacher. Preferably in a room with good sound insulation. (Strong Recommendation – Low-Quality Evidence)
II Speech assessment and therapy (if necessary) should be conducted as early as possible. (Strong Recommendation – Low-Quality Evidence)
III Genetic testing in children with SSD without other clinical signs. (No Recommendation)
IV Congenital UHL can cause a number of developmental problems in childhood and should be identified as early as possible. (Strong Recommendation – Moderate-Quality Evidence)
V CND and cCMV are the main causes of congenital UHL and should be prioritized in investigation. (Strong Recommendation – Moderate-Quality Evidence)
VI Patients with congenital SSD due to cCMV may develop late-onset bilateral hearing loss. Therefore, these patients should be monitored. (Strong Recommendation – Low-Quality Evidence)
VII The diagnosis of cCMV can be made only by collecting samples up to 4 weeks after birth using a PCR assay (blood, saliva, or urine). Samples collected later have low diagnostic value. DBS has been an option, although not yet very sensitive, but it can be used for children with suspected cCMV who have not undergone other tests at the appropriate time. (Weak Recommendation – Moderate-Quality Evidence)
VIII Imaging is critical for the diagnosis and treatment of children with SSD due to the high risk of CND. (Strong Recommendation – Moderate-Quality Evidence)
IX The contralateral ear should always be monitored for the risk of late-onset hearing loss. (Strong Recommendation – Low-Quality Evidence)
X Children with congenital SSD should undergo hearing assessment regularly or if there is any evidence of previously established language deterioration. (Strong Recommendation – Low-Quality Evidence)

### Treatment of unilateral hearing loss in children

The information provided to parents of children with SSD and UHL regarding treatment options varies considerably. Some health care professionals even minimize the negative impact of SSD on child development, leading to uncertainty in clinical decision-making.^99^ Late diagnosis may occur due to progressive hearing loss or false negatives in hearing screening. Children with SSD respond to sounds through their better ear and, although language development may be delayed, they often communicate effectively in some situations. Treatment options for UHL and SSD have expanded in recent years – CROS systems, BCDs, and CIs. It is important to note that CROS systems and BCDs are unable to provide any binaural hearing benefits. This means that speech discrimination in noisy environments and sound localization remain largely impaired.^100^

#### Contralateral Routing of Signal systems

Conventional treatments for hearing rehabilitation of patients with SSD typically involve rerouting the auditory signal from the affected ear to the unaffected ear. CROS systems are used when no benefit is expected from fitting amplification to the ear with hearing loss, which is the case of patients with SSD. These devices offer a non-surgical approach and represent the least invasive solution available. CROS hearing aids consist of a microphone and transmitter in a hearing aid worn on the impaired ear, which transmits sound to the functioning ear via a wire or wirelessly.^101^ In cases of asymmetric hearing loss (sensorineural, conductive, or mixed), the aid on the better hearing ear can also provide amplification in addition to the CROS input, creating a configuration known as bilateral CROS (BiCROS). BiCROS is recommended for individuals with mild-to-moderate hearing loss in the better hearing ear.^102^

Evidence suggests that CROS hearing aids are successful in reducing the negative effects of acoustic head shadow and improving sound awareness and signal-to-noise (S/N) ratio when sounds are directed toward the affected ear.^103^, ^104^, ^105^ The unobtrusive design of current wireless CROS and BiCROS hearing aids is certainly an additional advantage, influencing the acceptance of these solutions. These devices are also easy to handle, especially by patients with SSD, and do not require sophisticated programming or fitting strategies.^104^ However, CROS hearing aids do not restore binaural hearing and cannot improve tasks that require binaural cues, such as sound localization abilities on the horizontal plane.^106^, ^107^ Furthermore, in listening situations where the signal of interest is in the better ear and noise is transferred through the CROS microphone from the affected side, there is a significant reduction in speech understanding.^104^, ^108^

Children with mild-to-moderate UHL can be fitted with hearing aids. However, in children with SSD, the use of conventional hearing aids is contraindicated as they do not provide benefits.^109^, ^110^ In the past, children with SSD were often observed without intervention if their school performance and language acquisition were unaffected. Pediatric CI users with bilateral deafness have an overall Health-Related QOL (HRQOL) similar to that of their peers with NH.^111^

#### Bone conduction devices – surgically implanted

BCDs are rerouting devices that transmit signals from the ear with SSD to the better ear via bone conduction, bypassing the air conduction pathway. Since the late 1970s, when the first hearing aids were implanted, many different implantable and non-implantable devices have been introduced. BCDs function similarly to CROS systems, but using a sound processor that is attached to the skull.

BCDs transform sound waves into mechanical vibrations through direct contact with the skull, facilitating transmission to the inner ear. Surgically implanted devices can be divided into 2 distinct types: percutaneous and transcutaneous. Transcutaneous devices can be further classified as passive or active. Percutaneous devices transmit vibrations through a percutaneous osseointegrated pin or abutment in the skull. This direct connection allows efficient signal transmission at all frequencies without skin or soft tissue impedance. However, complications such as skin reaction, granulation tissue formation, keloids, and soft tissue infection are not uncommon.^112^, ^113^

Passive transcutaneous devices consist of a device, similar to a percutaneous device, and an external part held in place magnetically that transmits vibrations transcutaneously to the implanted device, keeping the skin intact. A major advantage of these devices is the lower rate of skin complications. However, sound attenuation caused by soft tissues may occur, reaching up to 25 dB at 6000–8000 Hz compared with percutaneous implants.^114^ They may also cause discomfort due to the magnetic force required to secure the processor in place.^115^ Symptoms can be relieved by reducing magnet pressure and limiting device use. However, excessive pressure can lead to skin necrosis.^116^

Active transcutaneous devices consist of an external audio processor (containing microphone, processor, and battery) and an internal system that houses the magnet, coil, and bone conduction transducer. Sound waves are transmitted electrically from the external device to the internal device using technology similar to that of CIs. Because the internal device is the one responsible for generating mechanical forces against the skull, skin attenuation is minimized, and the magnet power can be considerably reduced.^117^

BCDs are more effective than CROS hearing aids for sound discrimination in noise and quiet.^118^ Patients may have improved speech discrimination in noise, with results ranging from -3.8 to -4.8 dB S/N ratio, depending on the location of the sound source.^119^ Users often experience reduced hearing benefit and gain over time. The discontinuation rate has reached 14% over a 50-month period, approximately 3% per year.^120^

BCDs can alleviate the head shadow effect and improve sound awareness on the affected side.^101^, ^121^ However, they cannot restore binaural hearing or improve sound localization ability.^101^, ^121^, ^122^ Stimulation of the contralateral auditory pathway is believed to play a role in suppressing tinnitus and may be an important factor for patients with this complaint.^122^, ^123^, ^124^

Discomfort with the device, concerns about sound quality, and subjective hearing impairment are among the main reasons for rejecting CROS hearing aids and BCDs.^125^, ^126^ Reluctance to use rerouting devices is also linked to changes in self-perception, aesthetic concerns, and the presence of negative stereotypes. Despite counseling (habits, work/social environment, expectations) and extensive preoperative testing of non-implantable BCDs, they are less effective than osseointegrated systems because patients experience signal attenuation through the skin and soft tissues, especially at high frequencies, and their viable duration of use may be limited due to the discomfort caused by the force required to secure them in place.^127^

Comparing an adhesive hearing system with a CROS hearing aid in a prospective randomized crossover study, Mertens et al.^128^ evaluated 17 patients with SSD who used each of these devices for 2 weeks, divided into 2 groups. The results showed that 70% of participants with SSD considered the adhesive system to be partially useful or better, with satisfaction levels similar to those using the CROS system. While sound localization improved with the adhesive system, there was no significant improvement in speech perception in noisy environments. Another study of 9 patients with SSD evaluated a device that transmits vibrations through the palate and showed improved speech recognition and overall QOL in both quiet and noisy environments.^129^

Chandrasekar et al.^130^ found statistically significant improvements in hearing outcomes, as measured by Children’s Home Inventory for Listening Difficulties (CHILD) questionnaire scores and hearing thresholds for speech in noise, using a BCD compared with no amplification. Similar results were found by other authors.^131^, ^132^ However, Chandrasekar et al. also observed a low level of adherence to BCD use. Despite improved audiological outcomes and CHILD scores, some patients chose not to use the devices. The major reason for rejection appears to be concern about cosmetic appearance and its impact on social acceptance.^133^

Surgically implantable BCDs with better sound quality are generally available for children over 5-years of age.^134^ The CROS system introduces an additional problem, which is the possible occlusion of the ear with NH.^134^ Very young children or those with a narrower External Auditory Canal (EAC) would have great difficulty using the CROS system. The auditory deprivation associated with SSD causes irreversible changes in the auditory cortex,^135^ which BCDs and CROS hearing aids cannot prevent because they do not provide hearing to the affected ear.^98^

#### Cochlear implantation

Used since the 1960s, CIs provide the most effective treatment to restore useful hearing in profound deafness and represent an option for patients with SSD to restore binaural hearing. CIs can improve speech understanding in quiet and noise, sound localization, and QOL in those affected by SSD.^136^ Interest in restoring binaural inputs by using CIs in patients with SSD has grown during the last decade, with several studies reporting improvements in speech recognition in noise, sound localization, and tinnitus control.^3^, ^5^, ^31^, ^137^^,^^138^

Initially suggested as a treatment for severe tinnitus in adults with SSD, CI indication has also been expanded to include rehabilitation of children and adults with SSD, regardless of tinnitus. In 2019, the Food and Drug Administration (FDA) approved CI for the treatment of SSD in children aged 5-years and older with profound sensorineural hearing loss in the compromised ear (PTA: 5, 1, 2, and 4 kHz of > 80 dB HL) and NH in the contralateral ear (PTA: 5, 1, 2, and 4 kHz ≤ 30 dB HL).^98^ Early evidence in children with SSD using a CI shows significant improvements in speech perception in quiet and noise, as well as in the accuracy of sound source localization.^19^ A systematic review of 119 children undergoing CI for SSD showed significant improvements in audiological and patient-reported outcomes after implantation.^5^ These results highlight the growing evidence supporting the subjective and objective benefits of binaural hearing in children with SSD after CI.

Children with bilateral hearing loss have better outcomes in language and speech recognition when they receive CI early than those who receive it later.^139^, ^140^ Longer duration of deafness has a negative impact on speech recognition.^141^ However, these factors should not be considered a limitation to CI in children with SSD. Favorable HRQOL benefits have also been reported in children with an older age at the time of surgery and longer duration of deafness.^142^

Pediatric CI users report a worse HRQOL at school and in the social environment than their hearing peers.^143^ In the study by Zeitler et al.,^142^ children with bilateral CI reported higher mean QOL scores in the general functioning and social-relations domains than their peers with UHL. This may be explained by the fact that parents of children with bilateral hearing loss likely perceive their child’s initial disability as subjectively worse than parents of children with UHL, so the HRQOL benefits may be perceived more strongly in children with bilateral hearing loss. In both cases, it can be understood that a child with UHL, unlike a child with bilateral hearing loss, is not completely dependent on the implant due to the ear with NH. Furthermore, social isolation for children with bilateral hearing loss from being unable to hear at all may be more profound than for children with UHL.

Central neural adaptations after CI in patients with SSD may differ from those in patients with bilateral hearing loss, potentially enabling the integration of electric and acoustic signals.^144^ While the full extent of this signal integration is still under investigation, it supports the subjective evidence of improved hearing ability. It has been demonstrated that children with asymmetric hearing loss can benefit from the restoration of binaural hearing with CI, without detrimental effects on the unaffected ear.^145^ Children undergoing CI for profound SSD report substantial reductions in listening effort after implantation, with demonstrated reversal of cross-modal cortical reorganization even after long periods of pre-implant deafness.^146^, ^147^

While single-word recognition with a CI alone is often used by clinicians as the primary outcome measure of CI benefit, the overall goal of CI in children with SSD is to provide benefits related to binaural hearing, specifically spatial hearing. While prospective studies of children with SSD receiving a CI are limited, case reports and retrospective studies have demonstrated the benefits of CI use for masked speech recognition.^85^, ^148^, ^149^ Long-term data suggest that the effects of CI use on hearing in noise for children with SSD are evident as early as 6-months after activation and are maintained for at least 24-months.^19^

CI is the only treatment for SSD that provides hearing to the affected ear and allows binaural auditory stimulation of the brain. Prospective clinical trials, case reports, and retrospective reviews have shown positive outcomes with CI use in this patient population. Studies have shown that children with SSD who listen with a CI experience improved speech recognition in noise^19^, ^150^, ^151^ and sound source localization^19^, ^148^, ^152^ compared with monaural listening or preoperative performance. Furthermore, studies of pediatric CI users with SSD generally report consistent device use.^31^, ^44^, ^153^

A study of children with UHL, using the Children With Cochlear Implants: Parental Perspectives Survey, found favorable responses toward CI in all 8 measured domains, with less strongly perceived benefits than children with sensorineural hearing loss undergoing CI only in the communication and general functioning domains.^111^ Furthermore, children with UHL and prolonged hearing loss or older age at implantation showed favorable benefits, which were similar to those of the younger cohort and the cohort with shorter duration of hearing loss.^154^

Assessing the benefits of CI (sound localization and speech perception in noise) in younger children with SSD can be difficult given limited cooperation and the need for an advanced level of task understanding. The identification of objective measures that do not require the active participation and cooperation of the child is desirable and necessary to better establish the benefits of CI in younger children. Therefore, the measurement of Cortical Auditory Evoked Potentials (CAEPs) for vocal stimuli could serve as a cortical biomarker of audibility and auditory processing efficiency in children with SSD who use a CI, which could be useful for management.^155^

Compared with their peers with NH, children with SSD are at a disadvantage with respect to word recognition in quiet, masked sentence recognition, and sound source localization. CIs can benefit children with SSD in unilateral and bilateral listening tasks, although their outcomes still lag behind those of their peers with NH. Patients with SSD have shown significant improvements in masked sentence recognition and sound source localization in the CI + NH condition, benefits that have been observed over 24-months of CI use.^3^

Consonant-Nucleus-Consonant (CNC) scores have been shown to stabilize after 6-months of CI use. After 24-months of CI use, word recognition in the CI ear ranges from 32% to 78% of correct words, with a mean of 58%.^19^ These findings are very similar to the results of Deep et al.,^149^ who reported a mean CI alone word recognition score of 56% correct for pediatric CI recipients with SSD and significant variability in scores (range, 4%‒88%). Beck et al.^156^ also found variable improvement in word recognition and attributed the variability to age at implantation.

Family counseling is a decisive step before CI indication in SSD, mainly because a certain proportion of these children have been shown to become nonusers. In addition to neurophysiological reasons, children may become nonusers due to other causes, such as lack of family support or emotional distress.^149^ The main feature linked to CI efficiency is represented by its daily use. Since optimal CI performance can only be achieved through relevant educational and family support,^157^ CI use may be negatively affected by the lack of such support, particularly if family expectations about CI outcomes are not met. Another factor that can eventually limit CI use (in terms of hours per day) is that children may experience subjective benefits from the device only in specific circumstances, such as at school.

In children with SSD who are at risk of progressive hearing loss in the better ear (eg, in cases of CMV), surgery before hearing deterioration in the better ear is recommended to minimize auditory deprivation and improve hearing outcomes. Children with SSD due to bacterial meningitis should be implanted promptly. CND is a contraindication to CI in SSD. Accurate diagnosis of CND is important given its high prevalence in children with SSD.^98^

#### Age at CI surgery

Earlier age at implantation is beneficial in children with bilateral hearing loss.^140^, ^158^, ^159^ UHL begins to impact auditory development at very young ages, suggesting that early intervention may be necessary to mediate these effects. Yang et al.^160^ evaluated infants with UHL at a median age of 4.4-months and found delayed early prelingual auditory skills in infants with SSD compared with children with NH. Kishon-Rabin et al.^161^ reported delays in the auditory behavior of infants with UHL compared with infants with NH, and Fitzpatrick et al.^162^ observed delays in auditory function in preschool children with UHL.

Imaging and electrophysiologic data indicate that UHL can lead to marked cortical reorganization,^163^, ^164^ and restoration of hearing can reverse some of these changes and restore binaural function.^153^ While restoration of binaural hearing can occur in listeners with long-standing hearing loss,^165^, ^166^ hearing loss early in auditory development may be more detrimental than loss acquired later in development.^167^, ^168^ Younger age at CI surgery takes advantage of auditory neuroplasticity, which declines after 7-years of age,^169^ and may prevent aural preference syndrome, which develops after years of unilateral hearing.^3^, ^146^, ^167^, ^169^

A systematic review of children with UHL undergoing CI found that duration of hearing loss ranging from 4 to 7-years was associated with improvement in both audiologic and patient-reported outcomes.^5^ The duration of hearing loss that may determine outcomes is likely related to central auditory development, during which neural plasticity is at the highest. Auditory deprivation has been shown to result in delayed maturation of the auditory cortex and to affect a child’s neurocognition.^170^ A study using P1 latency as a measure of the maturity of central auditory pathways to evaluate children with congenital deafness using a CI^169^ found that the most sensitive period is up to 3.5-years of age, but neural plasticity exists up to 7-years of age, after which there is a marked decline.^169^

A systematic review assessed CI outcomes in children implanted for SSD, specifically addressing the child’s age at the time of surgery and duration of deafness, and found that the mean age of nonusers was higher than the mean age of daily device users.^5^ Also, a shorter duration of deafness was associated with improved outcomes. Beck et al.,^156^ in a series of 10 patients, determined that children with congenital deafness implanted after 4-years of age demonstrate poorer performance than children implanted before 4-years of age in SSD. Arndt et al.^171^ evaluated 36 children with SSD treated with CI – 20 had congenital, 7 had perilingual (0–4 years of age), and 9 had postlingual deafness (> 4-years of age). Children with congenital SSD showed better results with a shorter duration of deafness (< 3-years) than those with a longer duration of deafness.

Other studies have shown that children and adolescents with SSD who undergo CI can show significant improvement in speech recognition testing postoperatively despite age at implantation older than 7-years.^151^ Thomas et al.^172^ reported objective benefits in speech perception and sound localization in 21 patients with congenital SSD implanted up to 11-years of age and found no difference in Speech, Spatial, and Qualities of Hearing Scale (SSQ) scores in patients implanted before 6-years of age vs. those implanted after 6-years of age.

Park et al.,^98^ in children with SSD, suggested that the use of these devices at an earlier age could be beneficial given the importance of neuroplasticity in CI outcomes. Polonenko et al.^153^ demonstrated a rapid improvement in cortical reactivity, measured by electroencephalogram, after a few months of device use in children who received a CI before 3.6-years of age. In contrast, brain reorganization in response to SSD could hamper central binaural integration after CI, 2-years after the onset of hearing loss.^170^, ^173^

Children with SSD are likely to benefit more from CI when surgery is performed early (<3-years of age)^98^, ^172^ due to further maturation of the auditory pathway and myelination processes that have been reported to begin before birth and then continue up to the fourth year of life.^144^, ^174^ However, data on these features remain inconsistent.

Huttunen et al.^175^ reported a lack of high-quality studies investigating the consequences and benefits of interventions for early-onset and congenital UHL. In a systematic review, Benchetrit et al.^5^ noted that the studies consist mainly of retrospective reviews and investigations with small, heterogeneous samples and inconsistent methods. Longitudinal, repeated-measures, controlled trials are needed to provide professionals and families with evidence-based information on how to care for children with SSD.

Arndt et al.^137^ described better performance in children with post-verbal UHL than in children with pre-verbal or congenital SSD, who had worse outcomes in verbal discrimination and sound localization. Rahne and Plontke^176^ demonstrated satisfactory results using CI in pre-verbal and congenital SSD. Central brain adaptations, which occur after CI in SSD, differ from those in individuals with bilateral hearing loss, and it remains unknown how patients with SSD and those rehabilitated by CI can integrate electric and physiological acoustic stimulation over time.^177^ Probst^178^ hypothesized the lack of development of central compensation mechanisms in children with SSD after CI. This fact, combined with the aforementioned considerations, makes the choice of CI rehabilitation in children with SSD quite complex.

Yaar-Soffer et al.^155^ assessed the neuroplasticity of the central auditory pathways after CI using CAEPs, demonstrating improved skills in these children. In particular, available data support the benefits of electric stimulation, including improved myelination and expanded neuronal connections within the auditory pathway.

Using electroencephalographic data, Sharma et al.^146^ demonstrated that, even after several years of unilateral auditory deprivation, children can eventually exhibit neuroplasticity patterns within the auditory cortex after CI. Therefore, late CI could eventually improve the processing of auditory information in the brain. Further studies are needed in this field.

Regarding potential effects of CI on tinnitus, the available data is still insufficient in the pediatric population;^137^, ^146^, ^149^, ^157^ therefore, it is not possible to draw a conclusion on this specific topic.

#### CI discontinuation rate

Another factor that should be evaluated as it may affect the outcomes of CI users is the duration of device use. Daily device use has been shown to influence CI performance in children with bilateral hearing loss.^179^, ^180^ Many studies have reported a mean of 7 h of device use per day in CI users.^3^, ^138^, ^153^, ^181^ An important reason for performing CI surgery as early as possible would be for children and their environment to become accustomed to using the CI and to avoid the social stigma that can be a key factor in discontinuation of use when CI surgery occurs during adolescence.^3^, ^172^ In addition to the difficulty of adjusting the adolescent’s expectations about surgical outcomes.^182^

Park et al.^179^ observed that, in children with SSD and CI, better word recognition was associated with more hours of daily CI use. Arndt et al.^171^ evaluated 36 children with congenital and acquired SSD treated with CI. After a mean follow-up of 4.75-years, 32 of the 36 children were using their CI regularly.

Magee et al.^183^ evaluated 27 children with SSD, with a mean age at CI of 7.8 years. The mean daily device use was 7.8 (SD = 3.0) hours/day, and 40.7% of children used Direct Audio Input (DAI) daily. There was no significant correlation between hours of CI use and CNC score. However, there was a significant improvement in CNC score associated with the use of DAI during rehabilitation (CNC 50.91% [yes] vs. 23.81% [no]), which remained significant when adjusting for age at CI, duration of deafness, and daily device use. Therefore, this study indicates that DAI may be a useful tool in the rehabilitation of these patients.

#### Effects of age at CI activation

While the American Academy of Otolaryngology-Head and Neck Surgery (AAO-HNS) has attempted to find the optimal age at implantation for children with SSD, contraindicating surgery in children older than 3-years,^98^ a systematic review has suggested that findings are not yet robust enough to make recommendations.^5^ Park et al.^3^ found significant benefits of CI use for children implanted between 3.5- and 6.5-years of age. However, the age range of participants was quite narrow, limiting the ability to observe age effects. Almost half of the study population had an unknown onset of deafness, precluding a meaningful consideration of the impact of duration of deafness on CI outcomes. Unfortunately, this is not uncommon for children with UHL and SSD. If hearing loss is not present or detected on NHS, it may not be noted until hearing screening at elementary school enrollment. This is problematic because the incidence increases from 0.1% at birth to 3%‒6.3% in elementary school.^184^, ^185^

Recommendations

XI CROS hearing aids can successfully reduce the negative effects of acoustic head shadow and improve sound awareness and S/N ratio when sounds are directed toward the affected ear, being an option especially for patients unwilling to undergo surgery. (Strong Recommendation – Low-Quality Evidence)
XII Given the risk of partial occlusion of the ear with NH, with loss of the monaural cues that are important for the child’s development, CROS hearing aids have limited indication in this age group. (Weak Recommendation – Low-Quality Evidence)
XIII BCDs are more effective than CROS hearing aids for sound discrimination in noise and quiet, being particularly indicated in children. (Weak Recommendation – Low-Quality Evidence)
XIV Sound localization abilities are largely dependent on binaural cues. CROS hearing aids and BCDs have restricted indications for these purposes because they do not rehabilitate the deaf ear. (No Recommendation)
XV CIs provide the most effective treatment to restore useful hearing in profound deafness, being the only option for patients with SSD to restore binaural hearing. (Strong Recommendation – Low-Quality Evidence)
XVI CIs can restore binaural hearing, producing significant improvements in speech perception, spatial localization of sound, tinnitus control, and overall QOL. (Strong Recommendation – Moderate-Quality Evidence)
XVII There is low-quality evidence to suggest that CI for children with congenital SSD may be hardly beneficial to children older than 3-years. This should be evaluated on a case-by-case basis. (Weak Recommendation – Low-Quality Evidence)
XVIII CI in children with SSD due to CND. (No Recommendation)
XIX In children with SSD due to cCMV, CI is more appropriate due to the increased risk of contralateral hearing deterioration over time. (Strong Recommendation – Low-Quality Evidence)
XX Family counseling is a decisive step before CI indication in SSD to adjust expectations and conduct therapy. (Strong Recommendation – Moderate-Quality Evidence)

## Unilateral hearing loss in adults

In adults, the most common cause of acquired SSD is idiopathic, with other potential causes including cholesteatoma and infections, cerebellopontine angle tumors, and more rarely head trauma, autoimmune diseases, and Ménière’s disease.^74^ The incidence and prevalence of SSD in adults are not well established.^186^ The prevalence of UHL is estimated at 7.2%.^187^ This encompasses all UHL severities and is not specific to SSD. Kay-Rivest et al.^188^ estimated the prevalence of SSD in the United States to be 0.11%‒0.14% (271,122–345,064 adults). Wie et al.^189^ assessed the social impact of SSD in adults and found that 93% of affected individuals believed that their hearing loss had a negative effect on social interactions.

The onset of SSD in adulthood is often sudden.^190^ Even a small asymmetry between the ears has the potential to impose difficulties in sound discrimination, especially in competing-noise situations.^191^ Consequently, SSD gives rise to substantial difficulties with listening in most everyday situations.^192^ It impairs the ability to understand speech in noise and to localize sounds and also limits awareness of sounds that are located on the side of the affected ear.^193^ These difficulties and their consequences for social and occupational activities can lead to feelings of annoyance, embarrassment, and depression.^108^ There are safety risks, such as not hearing a vehicle or bicycle approaching from the deaf side, as well as a high cognitive load required to process auditory information.

SSD is often associated with several symptoms that can significantly impair a person’s daily life. Tinnitus is a common problem. Between 54% and 84% of adults with SSD experience disabling tinnitus.^189^, ^194^ Patients may also experience hyperacusis,^195^ aural fullness, and changes in the vestibular system, especially in cases of cochleovestibular impairment such as in Ménière's disease.^196^, ^197^

In individuals with NH, unilateral auditory stimulation causes activation predominantly in the contralateral auditory cortex (contralateral dominance).^198^, ^199^ UHL with disruption of binaural inputs results in brain reorganization with weakening of the representation of the deprived ear and strengthening of the representation of the intact ear (aural preference syndrome).^14^, ^135^ Brain reorganization is detectable 5-weeks after onset of SSD in adults. Functional MRI studies have demonstrated that brain reorganization stabilizes after 1-year^200^ and that the dominance shift also affects the nonprimary auditory cortex.^201^

Binaural cues are essential for the localization and perception of target sounds, especially in the presence of noise. Depending on the location of the sound source, as the 2-ears are physically separated on either side of the head, the signals reaching each ear may differ in time of arrival (interaural time difference) or intensity (interaural level difference). Other advantages of binaural listening include the head shadow effect (which causes listeners to focus on the ear with a better S/N ratio), binaural summation (a special case of binaural redundancy), and squelch effect (which allows the brain to suppress competing noise for better speech perception in noise).^202^

SSD has been associated with increased anxiety levels, difficulty communicating in the presence of background noise, and decreased self-esteem.^203^, ^204^ Difficulty communicating in noisy environments leads patients with SSD to withdraw from social situations, impacting personal and professional relationships. The impact of SSD also extends to general well-being. People with UHL are more likely to report health problems, dissatisfaction, and loneliness than those with NH. Even with the use of hearing aids, patients with SSD often experience a decline in HRQOL.^102^, ^205^

### Treatment of unilateral hearing loss in adults

In a systematic review of studies on the effectiveness of different interventions (CROS, BCD, and CI) in SSD, Kitterick et al.^206^ identified that the results have been somewhat biased toward assessing functional impairments for which measures are available and widely used, such as testing of speech perception in noise. However, the difficulties imposed by SSD can also affect the individual’s psychological and social well-being.^108^ Therefore, outcomes that assess the impact on an individual’s overall health and well-being are also relevant and potentially important.^207^ Health care users express uncertainty about the choice of treatment options for SSD often due to a lack of clarity about their benefit.^208^

The need for harmonization of assessment methods across SSD intervention trials is well recognized.^1^ These include daily device use, pure tone audiometry, free-field testing of speech perception in noise and sound localization, the SSQ scale, the Health Utilities Index Mark 3 (HUI-3), and if applicable, the Tinnitus Functional Index.^209^, ^210^, ^211^

#### CROS systems and BCDs

The indication of CROS systems for adults is adopted worldwide^212^ because this modality can be used promptly, without the need for surgery. Despite the restricted indication in children, it has proven to be a viable option for adults.

Contralateral routing of sound was first achieved by connecting a hearing aid microphone on the side of the deaf ear to a hearing aid on the side of the NH ear.^213^ A similar result can now be achieved via wireless communication between 2 behind-the-ear devices.^108^ Evidence suggests that CROS hearing aids are successful in reducing the negative effects of acoustic head shadow and improving sound awareness and S/N ratio when sounds are directed toward the affected ear.^100^, ^213^ In patients with tinnitus, the outcomes of their fitting are not encouraging.^206^ Patients may therefore need counseling to form appropriate expectations about situations in which benefit may be obtained and those in which the use of a rerouting device may be counterproductive to listening.

Regarding the effects of BCDs on QOL, a systematic review and meta-analysis found that BCDs are associated with significant improvements in hearing-related QOL in adult patients, whereas no difference was found in the measures of generic QOL as assessed by the HUI-3.^214^

Evidence suggests that rerouting devices can improve speech perception in noise. Statistically significant benefits to speech perception were found only when the S/N ratio was more favorable in the impaired ear than in the normal ear. In this situation, a rerouting device increases the S/N ratio in the normal ear by overcoming the head shadow effect.^215^ There is heterogeneity in the benefits of BCDs, with improvements in the S/N ratio ranging from 0.4^215^ to 4.4 dB.^107^ Therefore, uncertainty remains as to whether the magnitude of the benefit would be clinically meaningful.

Sound localization abilities are largely dependent on binaural cues,^216^ and rerouting devices do not restore 2-eared hearing. Some have speculated that these devices may provide cues that allow the listener to distinguish sounds on the left from sounds on the right (lateralization) by distorting the spectral content of sounds transmitted via the device. There is no evidence to suggest that devices that reroute sounds to the ear with NH provide reliable cues to support spatial hearing.^206^

#### Cochlear implantation

Oh et al.^217^ conducted a systematic review and meta-analysis of 50 studies involving 674 adult patients with SSD undergoing ipsilateral CI and showed statistically significant improvements in speech perception, tinnitus reduction, sound localization, and global and disease-specific QOL. Similar results were found in other systematic reviews conducted by Van Zon et al.,^218^ Peter et al.,^219^ and Levy et al.^220^ Karoui et al.^221^ showed that restoration of binaural hearing results in reversal of the abnormal pattern of cortical lateralization in individuals with UHL, resulting in improved spatial hearing.

Adult CI users with SSD have shown significant improvements in their sound localization abilities within the first few weeks of activation, and this improvement has been sustained after 1-year of CI use. Furthermore, localization accuracy and consistency continued to improve over the 5-year follow-up period after activation.^222^ Advanced age is not a contraindication to CIs, which have risks and individual performance outcomes for patients of advanced age similar to those observed in younger adults.^223^

Several studies have demonstrated a clear negative correlation between duration of deafness and CI performance in individuals with SSD due to the effect of prolonged monaural hearing on the auditory pathways.^224^ However, in adults with SSD, prolonged deafness should not be the only contraindication to CI. Rader et al.,^225^ in a retrospective analysis of 36 adults with postlingual deafness, found a more favorable result in speech perception 12–36 months after CI activation in patients with duration of deafness < 400-months. For those with a longer duration, success is limited but still possible. Nassiri et al.^226^ found no difference in speech perception among patients with SSD using a CI, regardless of whether their deafness had lasted more or less than 10 years.

Studies comparing rerouting technologies vs CIs in adults with SSD have shown that CIs significantly improve sound localization and that CI users perform equally or significantly better on measures of speech recognition in noise and subjective benefit.^227^, ^228^

The importance of intensive auditory training in CI users with SSD has been highlighted aiming to promote the perceptual integration of the electrically stimulated ear with the dominant ear, thus providing individuals with binaural hearing and optimizing outcomes.^1^, ^148^, ^229^ Further studies are needed to evaluate the effectiveness of optimal auditory training methods and to determine the recommended timing for individuals with SSD using CI.

CIs can also improve sound localization for individuals with contralateral residual hearing.^230^ Arnd et al.^137^ assessed sound localization before and after CI and identified a statistically significant improvement. Although CI undoubtedly restores some form of binaural hearing, it remains unclear whether it can restore spatial hearing in individuals with SSD and over what time scale.

Recommendations

XXI The indication of CROS systems for adults is most commonly adopted because this modality can be used promptly, without the need for surgery, and most of these devices can be transformed into BiCROS hearing aids for use in cases of deterioration of an initially normal ear. (Weak Recommendation – Low-Quality Evidence)
XXII BCDs are associated with significant improvements in hearing-related QOL in adult patients. (Strong Recommendation – Moderate-Quality Evidence)
XXIII Adult patients with SSD undergoing ipsilateral CI have statistically significant improvements in speech perception, tinnitus reduction, sound localization, and QOL. (Strong Recommendation – Moderate-Quality Evidence)
XXIV Despite the negative correlation between duration of deafness and CI performance in individuals with SSD due to the effect of prolonged monaural hearing on the auditory pathways, evidence is lacking to determine the threshold duration of auditory deprivation to indicate CI in this population. (Weak Recommendation – Low-quality Evidence)

## SSD in temporal BONE TUMORS

Hearing rehabilitation in patients with ear tumors and SSD is a controversial topic. While there is a need for restoration of binaural hearing, this is never a priority in the setting of benign and malignant tumors of the temporal bone and skull base. Therefore, when feasible in this scenario, forms of hearing rehabilitation such as CROS systems, BCDs, and CIs can be offered. Selection will be determined by factors such as patient choice, tumor features, and requirements associated with the proposed treatment for the tumor. In this section, we will discuss potential indications for surgically implantable systems and the scientific literature available on the topic, which is based on case series and systematic reviews with a low level of evidence.

### Sporadic vestibular schwannoma

SSD is an important symptom of sporadic Vestibular Schwannoma (VS). Because most patients with VS experience hearing loss, hearing preservation and rehabilitation are part of VS management. If there is profound UHL related to VS and/or resulting from treatment (surgery and/or radiosurgery), several treatments can be employed, such as CROS hearing aids, BCDs, and CIs. CI is the only treatment with the potential to restore hearing in these patients, along with restoration of binaural hearing.

### Cochlear implantation

#### Intraoperative assessment of nerve integrity

CI may be useful in cases where the functional integrity of the cochlear nerve is preserved. Some surgeons rely only on the subjective assessment of the anatomical preservation of the cochlear nerve to decide whether CI could be performed. However, there are several objective measurement tools to assess cochlear nerve function, such as electrically evoked Auditory Brainstem Response (eABR). This monitoring can provide objective information on functional preservation of the cochlear nerve during tumor resection.

Electrical stimuli can be delivered using multiple methods. A rounded bent-tip (“hockey stick”) electrode can be placed in the round window niche and used to stimulate the cochlear nerve.^231^ This method is comparable to the promontory stimulation test. However, promontory stimulation relies on the subjective auditory sensations experienced by the patient in response to electrical stimulation and has been shown to have poor prognostic value considering speech performance with CI. The use of this method as a screening tool for CI was subsequently limited to cases of cochlear malformations. In contrast, eABR with the “hockey stick” electrode provides objective results and has proven to be much more useful in determining the excitability of the auditory nerve pathway – especially in patients with inconclusive preoperative audiological tests.^232^ Therefore, eABR with the “hockey stick” electrode can be performed in patients under local anesthesia, mainly to assess prognosis.

In addition to round window eABR, there is also the possibility of stimulating the auditory nerve via the cochlea. A test electrode can be used to provide appropriate stimuli for eABR measurements. In the case of CI after VS resection, the functional integrity of the cochlear nerve can be confirmed prior to CI insertion using an intracochlear test electrode.^233^ Unlike the CI, the intracochlear test electrode only has 3 contacts at an array length of 18 mm and a separate ground electrode. This device is subsequently removed and discarded after measurement. These evoked neural responses are measured using electrodes placed on the patient’s head. There is also the possibility of near-field ABR recordings using an electrode placed directly on the auditory nerve (cochlear nerve action potential) or using a surface electrode located on the dorsal cochlear nucleus (dorsal cochlear nucleus action potential).^234^ Despite these advances, there is no definitive evidence that eABR is a reliable tool for assessing cochlear nerve function during VS resection.^235^ Of all the various eABR features to be interpreted, in the case of VS resection, simply the presence of wave V has proven to be a reliable indicator of cochlear nerve function. A loss of wave V amplitude greater than 50% or an increase in wave V latency greater than 1 ms may act as warning signs, although no predictive value of these factors for postoperative hearing outcome has been confirmed in the literature. The presence of a clearly detectable eABR wave V after tumor resection may prompt consideration of CI. There are no general recommendations on what actions should be taken in cases where there is partial or complete loss of eABR signal. Systemic and local factors may influence the eABR signal during surgery. For example, systemic or local hypotension may contribute to a loss of amplitude and increase in wave latencies. When complete loss of an eABR wave V occurs, CI may not be successful. However, there are reports of patients with negative eABR responses undergoing CI who later achieve auditory perception.^236^ A few patients with a preserved eABR wave V may not achieve auditory perception following CI. It is essential to provide appropriate preoperative counseling and manage patients’ expectations, as well as to consider that the final decision should be well individualized for cases of sporadic VS and SSD.

#### CI outcomes in sporadic VS

The first prospective study assessing the audiological outcomes of CI after translabyrinthine resection of sporadic VS was conducted by Rooth et al.^237^ Seven patients met the inclusion criteria (small tumors, surgical feasibility, and willingness to participate in postoperative rehabilitation) and underwent simultaneous CI and VS resection; the main determinant for CI was the surgeon’s subjective perception of an intact cochlear nerve immediately after VS resection. Five patients had auditory perception at the time of activation, whereas 2 remained without auditory perception. All patients experienced subjective improvement in sound localization, speech perception in noise, and tinnitus severity. However, the study also demonstrated that patients with better outcomes achieved with VS have worse outcomes than patients undergoing CI for idiopathic UHL.

Sanna et al.^238^ conducted a prospective study with the largest series of patients with normal contralateral hearing undergoing CI insertion simultaneously to sporadic VS resection. A total of 13 individuals underwent VS resection by means of a modified translabyrinthine approach with cochlear nerve preservation and simultaneous CI and showed a slight improvement in the mean PTA at low frequencies, which was not statistically significant (*p* >  0.05). Furthermore, PTA, disyllabic word recognition, sentence recognition, and common phrase comprehension improved at the second control, but this improvement was also not statistically significant (*p* >  0.05). Regarding CI use, 90% of patients reported using the device between 5 and 7 days per week. The median subjective satisfaction score on a 10-point scale was 8. The main limitation of the study was failure to perform an intraoperative assessment of cochlear nerve function.

Conway et al.^239^ showed similar results in their prospective study in terms of improved speech discrimination in noise, improved sound source localization, and decreased tinnitus. Tadokoro et al.^240^ conducted a systematic review of 16 articles published from 1995 to 2017, including 45 patients. The postoperative auditory outcomes were as follows: Speech dDiscrimination Score (SDS) of 56.4% (SD = 27.6%) (vs. preoperative SDS of 30.0% [SD = 40.0%]); PTA of 28.8 (SD = 8.3) dB (vs. preoperative PTA of 79.8 [SD = 32.7] dB); and Arizona Biomedical Institute Sentence Test (AzBio) score of 75.0% (SD = 14.3%). Of the 7 patients with tinnitus complaints, 5 reported subjective improvement in symptoms. Thompson et al.^241^ conducted a systematic review of concurrent CI and resection of sporadic VS and Neurofibromatosis type 2 (NF2). Approximately 85% of patients had auditory perception with their CI and 75% had high-to-intermediate performance in terms of audibility (AzBio score of 72% in NF2 vs. 70% in sporadic VS). Most patients considered to be low performers still reported some subjective benefit from CI compared with preoperative measures. The main predictor of audiological outcome was tumor size (*p* = 0.018).

Longino et al.^242^ assessed CI outcomes in small, non-operated VS. Seven patients with VS volumes ranging from 0.11 to 1.02 cm^3^ were implanted. All patients were on imaging follow-up for 2-months to 7-years. Monosyllabic word recognition improved in all patients (from 6% to 55%), with stable performance at a follow-up of at least 12-months.

The level of evidence is low because, to date, only case series with small sample sizes are available. A few systematic reviews have been published in recent years on CI outcomes after sporadic VS resection, but the data from these studies are highly heterogeneous due to the large number of case reports included and the lack of standardized tools to assess hearing performance after implantation. There is likely publication bias in these successful cases. Only a few studies are prospective, and none have a comparative design. Furthermore, high variability in follow-up time, auditory test batteries, and preoperative measurements is responsible for meta-analyses with high heterogeneity.

#### BCDs and CROS systems

The effectiveness of hearing rehabilitation with BCDs in patients with VS remains controversial. There are few published studies on hearing rehabilitation with BCDs in patients with SSD after VS resection. Bouček et al.^243^ assessed the audiological outcomes of 16 patients who accepted to undergo BCD implantation and found a significant improvement in sentence discrimination at both 6-week (64.0%) and 1-year (74.6%) follow-up. This improvement was noted in situations where the sentences came from the side of the affected ear, with contralateral noise of -5 dB S/N ratio. Another study evaluated subjective hearing handicap in patients with SSD after VS surgery and the effect of the BCD on the test band. Twenty-six patients were included and showed a mean improvement in speech discrimination in noise of 15%. However, only 50% of patients agreed to undergo BCD implantation after the test.^107^

Many studies comparing CROS and BCD technologies are available, but unfortunately none are specifically focused on patients with VS. Therefore, studies with long-term follow-up are lacking to determine the actual benefits in this population.

#### Intracochlear schwannoma

Aschendorff et al.^244^ published the results of 8 patients in a retrospective study. While no specific data on preoperative or postoperative hearing parameters were included, they reported that patients with simultaneous CI placement tend to have more favorable rehabilitation outcomes than those receiving a CI in two-stage, sequential surgery. Speech understanding obtained in their cohort of implanted patients was reported to be comparable to that of other patients with SSD of different etiologies.

#### Irradiated vestibular schwannoma

Tian et al.^245^ conducted a systematic review of 14 studies of patients with sporadic VS and NF2, both irradiated. Hearing outcomes were considered poor in 27% and excellent in 19%, with a mean follow-up of 19-months. Outcomes were considered intermediate in 70% of patients.

Recommendations

XXV CI is a treatment option for SSD associated with sporadic VS, especially in the presence of disabling tinnitus and preserved cochlear nerve function. (Strong Recommendation – Low-QualityEvidence)
XXVI Intraoperative eABR measurements are suggested to help inform decision-making on CI candidacy after VS resection. (Weak Recommendation – Low-Quality Evidence)
XXVII Despite not restoring binaural hearing, CROS systems and BCDs may be indicated in patients with VS-related SSD. It is recommended to perform preoperative tests with BCDs. (Strong Recommendation – Low-Quality Evidence)

#### Paragangliomas

After undergoing surgical resection with preservation of the EAC anatomy, patients may suffer from sensorineural, conductive, or mixed hearing loss and benefit from conventional personal hearing amplification devices.

In patients with SSD and maximal conductive hearing loss as a result of treatment, bone-anchored hearing aids may be a viable option. Patients undergoing subtotal petrosectomy with blind sac closure of the EAC have a large conductive hearing loss. These patients with maximal conductive hearing loss may benefit from BCDs.^246^ In these cases, considering imaging artifacts and a possible need for postoperative imaging monitoring, percutaneous hearing aids can be used.

Cases of profound unilateral sensorineural hearing loss due to temporal bone paragangliomas may occur for a variety of reasons. These situations are associated with intracochlear erosion secondary to tumor expansion, invasion of the IAC, or brainstem compression. In these situations, postsurgical patients are rarely CI candidates. However, in selected patients with progressive hearing loss and preserved cochlear anatomy, CI may be a viable option. CI was reported in a young patient treated surgically for bilateral paragangliomas. The implanted side was treated via canal-wall-up mastoidectomy without blind sac closure of the EAC. Despite good initial postoperative performance, the patient progressed with worsening of hearing due to cochlear ossification.^247^ A case of successful CI in patients with post-radiation paraganglioma has been reported.^248^

While CI is rarely indicated in patients operated on for paragangliomas, it may be feasible in well-selected cases.

#### Imaging artifacts

Regardless of etiology, imaging follow-up is a consideration in all patients requiring assessment of CIs and transcutaneous bone-anchored hearing aids after treatment of temporal bone tumors.

While it is currently possible to perform MRI up to 3.0 T, distortion artifacts are present. There are methods to reduce these artifacts, especially so as not to impair lesion visualization, thus allowing regular imaging surveillance in patients receiving a CI. Proper head positioning, magnet placement at a distance greater than 6.5 cm from the EAC, use of spin-echo sequences, and fat suppression techniques can reduce the size and shape of MRI artifacts.^249^, ^250^ In any case, it is important to take into account imaging artifacts with CIs and transcutaneous hearing aids and potential difficulties in MRI follow-up.

Recommendations

XXVIII In patients with profound unilateral sensorineural hearing loss and neoplasms that require long-term imaging follow-up, the harm caused by the presence of imaging artifacts during follow-up should be considered when deciding on the use of CIs and transcutaneous bone-anchored hearing aids. (Weak Recommendation – Low-quality Evidence)

### Limitations

The lack of prospective controlled trials and the exclusion of patients who provided incomplete data raise concerns about the potential for selection bias to influence the observed effects. No recommendations for the management of adults with UHL can be based on current evidence. The primary recommendation is that randomized controlled trials be conducted to compare devices. If further cohort or case-control studies are undertaken, they should be well designed, adequately powered, and prospectively planned with detailed inclusion criteria, and should recruit patients from multiple sites.

## Conclusion

The etiology of SSD can influence device performance and usage. Patients with SSD secondary to cCMV may have poorer performance and variable outcomes compared with other implanted patients. Additionally, cochlear nerve hypoplasia is reported in a variable percentage of children with SSD,^251^ and those affected are less likely to respond to electrical stimulation generated by the CI. MRI is always essential to identify this condition and to avoid misleading indications and management.^176^

Decision-making for patients with SSD is complex and multifactorial. The lack of unanimous consensus on outcome domains and measurement tools hinders a proper comparison of different treatment options. Rerouting devices (CROS systems and BCDs) can alleviate the head shadow effect and improve sound awareness and S/N ratio in the affected ear. However, they cannot restore binaural hearing. Because CROS hearing aids are not surgically implantable, they are the least invasive option and can be tested primarily in adults. Among BCD options, percutaneous devices usually involve skin complications, while passive transcutaneous devices avoid these problems but can cause discomfort owing to the magnetic force required to secure the processor in place. Active transcutaneous BCDs address these drawbacks but require a larger implantation site. Reluctance to use rerouting devices is also associated with changes in self-perception, aesthetic concerns, and negative stereotypes. In malignant temporal bone tumors, especially those that have undergone adjuvant radiotherapy, the occurrence of osteoradionecrosis may compromise osseointegration with the use of percutaneous BCD.

CIs are distinguished by their ability to restore binaural hearing, producing significant improvements in speech perception, spatial localization of sound, tinnitus control, and overall QOL. However, CIs are not recommended in cases of CND, a relatively common cause of congenital SSD. Preoperative anxiety contributes to the rejection of BCDs and CIs. Therefore, when deciding on treatment strategy, it is crucial to carefully consider the duration and etiology of deafness. However, it is also necessary to identify the family’s needs and goals.

In addition to treating SSD, the concern for maintaining the health of the normal ear is also important. It is essential that the patient and family are informed of the importance of annual audiometric monitoring, the use of ototoxic agents, and the need to avoid exposure to noise (occupational and recreational) to prevent hearing deterioration.

## Funding

The authors have no financial relationships relevant to this article to disclose.

## Conflicts of interest

The authors declare no conflicts of interest.
